# Research on the impact mechanism of Hubei traditional festival music activities on collective belonging and psychological safety in rural residents

**DOI:** 10.3389/fpsyg.2026.1757642

**Published:** 2026-02-10

**Authors:** Qi Gao, Yu Xia

**Affiliations:** School of Music and Dance, Huaihua University, Huaihua, China

**Keywords:** cultural confidence, Hubei traditional festival music activities, life satisfaction, psychological sense of safety, sense of collective belonging

## Abstract

This study takes rural areas in Hubei province as a case, using 378 valid samples and employing structural equation modeling for empirical testing. The results show that Hubei traditional festival music activities can promote the development of cultural confidence among rural residents. Additionally, cultural confidence positively influences the sense of collective belonging and psychological sense of safety. The sense of collective belonging also enhances psychological safety. Moderation analysis reveals that life satisfaction positively moderates the effect of cultural confidence on both collective belonging and psychological safety. Theoretically, this study expands the research on the social functions of traditional festival music and enriches the cross-level relationship between cultural confidence and psychological safety. Practically, the research provides a basis for local governments and cultural management departments to foster social cohesion and sustainable development in rural communities.

## 1 Introduction

Hubei traditional festive music, deeply embedded in the region’s cultural heritage, is more than an art form; it is a vital social mechanism that plays a pivotal role in regulating emotions and promoting psychological well-being (Wang and Thotham, 2025; Hu et al., 2024). In the face of globalization and rapid modernization, many traditional practices—such as Hubei’s festive music—are at risk of disappearing (Mingfeng and Pattananon, 2024). As rural populations face increasing psychological pressures, especially with the fragmentation of traditional social structures and communal ties, the role of these cultural forms in fostering emotional belonging and psychological safety becomes crucial (Zhou and Gu, 2025). Re-examining these practices in the context of contemporary society, particularly their capacity to reinforce both individual and collective identity, emotional cohesion, and resilience, has become an essential area of inquiry (Zhang et al., 2024).

For generations, music has been central to rural life in Hubei, integral to daily routines, festivals, and community events (Zhang, 2017). The distinct rhythmic patterns and melodic structures of Hubei’s folk music reflect the historical, geographical, and cultural nuances of the region (Feiyan et al., 2024). Beyond mere cultural expression, music functions as a medium through which individuals come together to collectively express emotions, build social bonds, and affirm communal identities (Jiayang Li and Su, 2024). This is especially evident during festive occasions, where music provides a shared space for cross-generational interaction and the reinforcement of social ties. These communal musical experiences nurture a profound sense of belonging, which is essential for the social fabric of rural communities (de-Miguel-Molina et al., 2021).

Psychologically, music has long been recognized as a vehicle for emotional resonance, fostering synchrony among individuals and reinforcing social identity (Shandil, 2024). Research in psychology suggests that engaging in collective musical activities—whether through singing, dancing, or playing instruments—enhances participants’ psychological safety by strengthening their connection to the group (Ning, 2023). In Hubei, where traditional festive music is central to community events, these experiences are instrumental in cultivating emotional security. Music offers an outlet for emotional expression, while also reinforcing communal bonds that are crucial for rural residents’ social stability and well-being (De Leeuw et al., 2022).

Beyond its emotional benefits, music also holds significant therapeutic potential (Gouk, 2017). Modern psychological research highlights music’s positive impact on mental health, including its ability to reduce stress, improve emotional regulation, and enhance well-being. In the context of Hubei’s traditional festive music, these therapeutic effects are amplified (Silverman, 2022; De Witte et al., 2021). By participating in collective musical experiences, rural residents can alleviate anxiety, achieve emotional relief, and navigate the psychological challenges of modern life. In areas with limited access to mental health resources, such as rural Hubei, music festivals serve as essential spaces for emotional healing and psychological comfort.

Moreover, music plays a vital role in reinforcing social support networks, a central tenet of social support theory. By fostering a sense of belonging and emotional connection, music strengthens communal bonds and facilitates informal support networks (Hallam and Himonides, 2022). In Hubei’s traditional music festivals, these networks are solidified through group participation and shared cultural practices. The social interactions that music encourages enable individuals to affirm their identities, establish emotional ties, and ultimately contribute to the cohesion of the social fabric (Chalton et al., 2023). This informal support system is especially crucial in rural areas, where isolation and limited access to mental health services exacerbate feelings of loneliness and vulnerability. By enhancing social cohesion, music offers a protective buffer against the mental health challenges faced by many rural residents.

The concept of psychological safety is particularly relevant in the context of rural communities, where economic shifts and migration often lead to feelings of disconnection and insecurity (Newman et al., 2017). Defined as a shared sense of trust and inclusion within a community, psychological safety is essential for individual well-being (Frazier et al., 2017). In Hubei, where traditional support systems are eroding due to economic pressures and migration, the role of festive music in promoting psychological safety is becoming increasingly critical (Wang et al., 2022). The communal nature of music festivals—characterized by collective participation and shared joy—creates an emotionally secure environment where individuals feel accepted and valued (Duffy, 2018). This sense of acceptance, in turn, fosters resilience, making music a crucial tool in safeguarding the psychological stability of rural residents amidst rapid modernization and social fragmentation (Laing and Mair, 2015).

Rural residents’ sense of collective belonging and psychological safety is foundational to community resilience and social cohesion (Singh et al., 2018). Despite challenges posed by urbanization and the erosion of traditional cultural practices, rural communities in Hubei continue to uphold cultural forms that bind individuals together. However, as migration, economic pressures, and social changes contribute to the decline of traditional cultural activities, the sense of belonging and emotional security provided by these practices is at risk (Petković, 2007). Therefore, it is vital to explore how Hubei’s traditional festive music influences the psychological experiences of rural residents and supports mental well-being and community stability. This study aims to fill a significant gap in existing literature by providing insights into how these cultural practices foster psychological resilience in rural populations.

Existing research has explored the role of music in preserving cultural identity and community cohesion (Gao and Shi, 2025; Kelmendi, 2024), but few studies have focused on its psychological impact, particularly in rural contexts. Much of the existing literature has concentrated on urban populations, often neglecting the specific psychological needs of rural residents (Jian et al., 2024). Additionally, studies on collective cultural practices like music festivals frequently treat them primarily as cultural or economic phenomena, overlooking their emotional and psychological significance (Chiya, 2024). This research shifts the focus by integrating cultural, psychological, and social dimensions, offering a holistic perspective on how traditional festive music contributes to rural well-being. In addition, this study specifically focuses on the life satisfaction of rural residents and examines it as a moderating factor. By examining the intersection of culture and psychology, this study provides novel insights into the ways in which traditional music promotes mental health and social stability.

This study also aims to explore how rural communities in Hubei can adapt their cultural practices to meet the challenges of the 21st century. Traditional music festivals are not mere relics of the past; they have the potential to be revitalized as tools for enhancing psychological well-being in contemporary rural settings. This research advocates for a rethinking of cultural preservation—not only as an act of safeguarding heritage, but as a dynamic practice that meets contemporary needs, especially in terms of promoting emotional belonging, community resilience, and psychological safety. By examining the role of traditional music in rural mental health, this study contributes to the broader conversation on how cultural practices can be integrated into modern psychological and social interventions.

Given the increasing challenges faced by rural communities—such as social fragmentation, mental health issues, and the erosion of cultural identity—the role of traditional music in supporting emotional well-being has never been more critical (Jin et al., 2024; Chen et al., 2021). While research on the connection between culture and mental health is growing, few studies have focused on how regional traditional music can specifically address the psychological needs of rural populations. This research aims to fill this gap, offering valuable insights into the contributions of Hubei’s festive music to rural residents’ sense of belonging and psychological safety. Additionally, the study provides practical recommendations for community-based interventions that incorporate traditional music as a tool for enhancing mental health and social cohesion.

In conclusion, this research offers a comprehensive exploration of the psychological benefits of traditional festive music in rural communities. It contributes both theoretically and practically by deepening our understanding of how cultural practices shape emotional belonging and psychological safety. By highlighting the mental health benefits of Hubei’s festive music, this study makes a significant contribution to the fields of cultural studies, community psychology, and social health. Furthermore, it advocates for the preservation and adaptation of these traditions, ensuring they continue to serve as a vital source of emotional support and community resilience in a rapidly changing world.

## 2 Literature review and research hypotheses

### 2.1 Hubei traditional festival music activities

**Figure 1:**
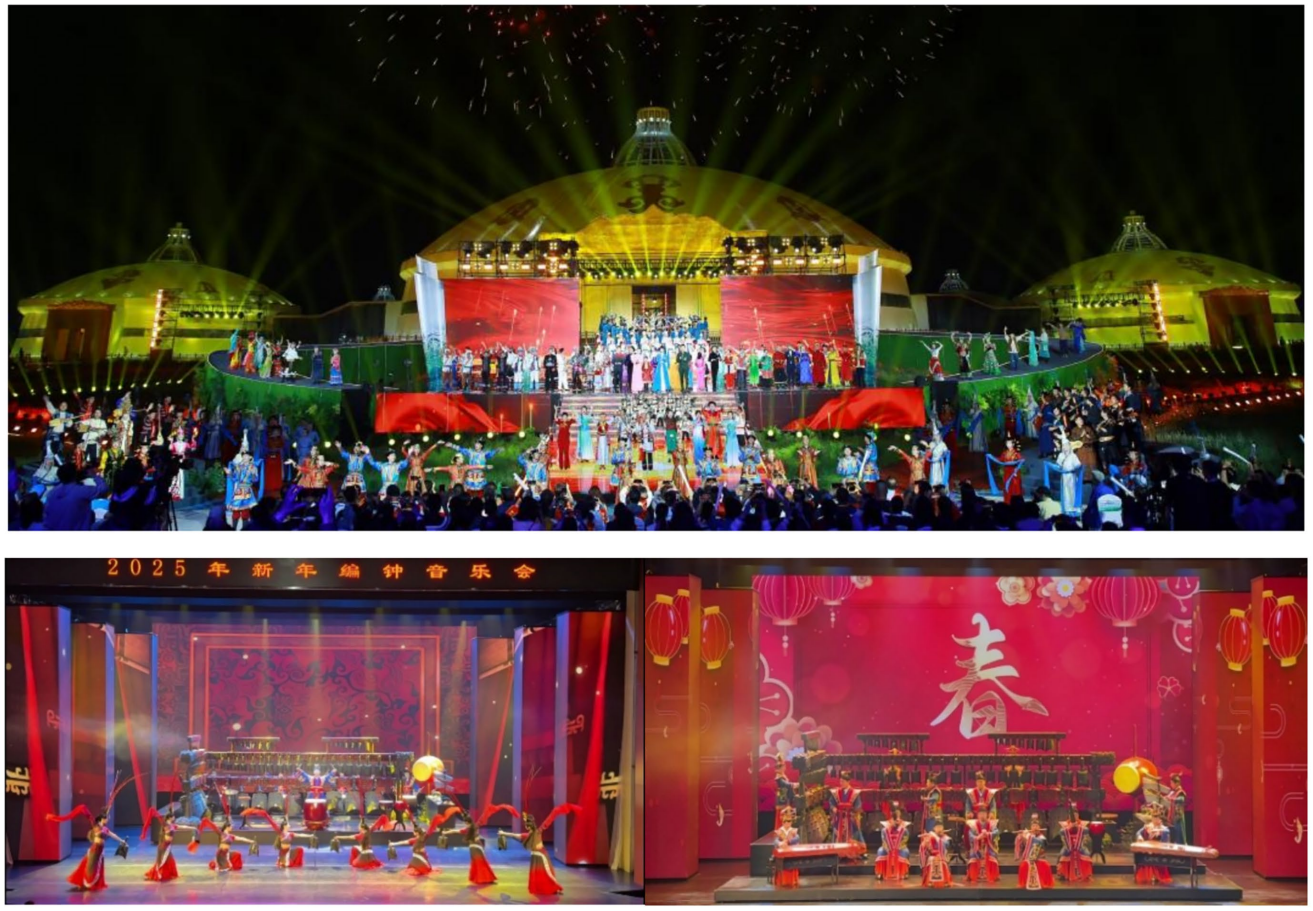
Hubei traditional festival music activities. Source: https://wlt.hubei.gov.cn/bmdt/dtyw/202507/t20250707_5714656.shtml; https://www.hbww.org.cn/xwdt/p/10496.html.

Hubei traditional festival music activities are deeply rooted in the region’s cultural heritage, with distinct characteristics influenced by both local traditions and the province’s unique geographic and ethnic diversity (see Figure 1).

These musical forms are primarily associated with important regional festivals, such as the Spring Festival, Dragon Boat Festival, and Mid-Autumn Festival, where music plays a central role in ritual performances, communal celebrations, and cultural preservation. The music is characterized by its use of traditional instruments, such as the pipa, erhu, gong, and drums, producing rich, vibrant sounds that evoke a deep connection to local traditions.

Additionally, Hubei festival music is often performed in specific modal scales, incorporating both pentatonic and heptatonic structures, which contribute to its distinctive melodic and harmonic characteristics. The rhythmic patterns in these performances are typically fast-paced and dynamic, creating an energetic atmosphere that encourages collective participation and fosters a sense of unity among villagers. These musical activities not only serve as a medium for cultural expression but also reinforce social bonds, thereby enhancing collective identity and psychological safety within rural communities.

### 2.2 Hubei traditional festival music activities and cultural confidence

The role of traditional festival music activities in rural communities goes beyond mere entertainment; these activities are deeply embedded in the cultural and social fabric of the community (Liu and Song, 2025). They serve as crucial mechanisms for the transmission of cultural knowledge, collective memory, and social cohesion, and function as a dynamic form of collective expression of cultural identity (Jian et al., 2024). In particular, music—often referred to as the “language of the soul”—is a powerful medium for the articulation of cultural narratives, values, and symbols (Wang, 2024). When individuals participate in or even observe these traditional music events, they reconnect with the deep historical and cultural roots of their community, experiencing an embodied connection to the past and reinforcing their cultural memories (Jiayang Li and Su, 2024).

From a psychological perspective, cultural activities provide a tangible platform for individuals to internalize, express, and reaffirm their heritage, in a manner that is both social and deeply personal. This process is aligned with the concept of cultural identity (Jiang et al., 2026), which posits that individuals form a sense of self through the recognition and validation of shared group characteristics, particularly cultural practices and traditions (Modood et al., 2025). The theoretical framework of cultural identity emphasizes the ways in which individuals’ identities are shaped by their affiliations with culturally significant practices (Li and Wood, 2016). Thus, the engagement with traditional music festivals allows individuals to engage in a process of meaning-making, through which they reinforce and solidify their connection to a particular cultural group. This, in turn, strengthens their personal and collective sense of cultural identity (Tao et al., 2020).

Additionally, the theory of social identity (Ellemers and Haslam, 2012) underscores the importance of cultural markers, such as music, in the formation of group identity. In rural Hubei, where traditional music plays a central role in community life, these festival activities represent one of the most significant avenues for individuals to affirm and celebrate their membership within their cultural community (Tekman and Hortaçsu, 2002). This ongoing engagement with cultural practices that have been passed down through generations deepens individuals’ emotional and psychological attachment to their community, further cultivating a sense of shared cultural pride (Bartleet, 2016; Mazlan et al., 2025).

The role of music in Hubei’s rural communities is not merely as a medium for cultural expression but also as an active process of social negotiation and identity construction. Music, as noted in symbolic interactionism (Carter and Fuller, 2015), acts as a communicative tool through which rural residents engage in social interactions that reinforce shared values and social cohesion. While this process is a key avenue for identity formation, it is essential to recognize that it occurs within a broader socio-cultural context that involves both historical and contemporary forces shaping these interactions.

By situating traditional music festivals within the framework of cultural heritage and social cohesion, I argue that these activities do not simply serve as passive reflections of tradition but are dynamic practices through which rural residents assert and redefine their cultural identity. This dynamic engagement with cultural practices, as illustrated by Zhong et al. (2025), allows for the continuous reaffirmation of one’s place within the community, reinforcing a sense of belonging and pride. Therefore, this discussion suggests that the promotion of cultural confidence is not solely an outcome of participation in these activities but also a process deeply embedded in the collective reinforcement of values, practices, and identity.

Hence, leads to the first hypothesis:

*H1*: Traditional festival music activities in Hubei can promote cultural confidence among rural residents.

### 2.3 Cultural confidence and sense of collective belonging

Relevant studies have shown that the establishment of cultural confidence lays a solid foundation for the development of collective belonging (Liebenberg et al., 2019). Social identity theory (Scheepers and Ellemers, 2019) suggest that individuals derive part of their self-concept from the groups they belong to. When cultural confidence is nurtured, individuals begin to view their community and cultural heritage as integral to their identity, enhancing their sense of attachment to the group. The act of celebrating cultural traditions, such as through music, fosters a collective sense of purpose, as shared cultural experiences reinforce the bonds between individuals (Rouchy, 2002; Liebenberg et al., 2019).

In rural Hubei, the reinforcement of cultural identity through traditional music directly facilitates social cohesion. The communal celebration of these traditions enables rural residents to reaffirm their shared values and history, strengthening their collective identity. The psychological mechanism here is rooted in the notion that as individuals feel more confident in their cultural background, they are more likely to invest emotionally in the social group that reflects that identity. Cultural confidence, therefore, provides the psychological framework within which individuals form and maintain their connection to their community (Li et al., 2025).

Research on collective efficacy has shown that when individuals feel a deep sense of confidence in their group’s identity and purpose, they are more inclined to engage in collective actions and emotionally invest in the welfare of their community (Rouchy, 2002). In this context, cultural confidence acts as a catalyst for strengthening group cohesion, fostering a shared responsibility and mutual care, which in turn enhances the sense of collective belonging. This mechanism of cultural confidence not only reinforces social ties but also nurtures a stronger community connection (Eaude, 2020). Drawing on these insights, we hypothesize that cultural confidence plays a pivotal role in fostering collective belonging among rural residents in Hubei, by influencing their sense of unity and social connectedness.

Therefore, leads to the second hypothesis:

*H2*: Cultural confidence can enhance collective belonging among rural residents.

### 2.4 Cultural confidence and psychological sense of safety

Psychological safety is defined as a state in which individuals feel safe to express themselves without fear of judgment, rejection, or harm (Newman et al., 2017). One of the key drivers of psychological safety is the perception that one’s identity is accepted and valued within the social environment (Pan et al., 2021). In the context of Hubei’s rural communities, where cultural identity is tightly linked to social cohesion, cultural confidence serves as an important precondition for individuals to feel secure in their social interactions (Walker et al., 2014).

When rural residents feel confident in their cultural identity, they are more likely to experience psychological safety within their community. This is because cultural confidence provides a psychological buffer against external threats or feelings of marginalization. The act of engaging in traditional music activities and celebrating cultural practices allows individuals to feel recognized and valued by their peers, which in turn reduces feelings of social insecurity (Frazier et al., 2017). Furthermore, from a self-affirmation theory perspective (Sherman and Cohen, 2006), individuals who have a strong sense of their cultural identity are less likely to experience anxiety in social settings, as they are assured of their value within the group.

Therefore, cultural confidence enables rural residents to engage more freely in social exchanges, as they feel emotionally secure and accepted by their community. In turn, this psychological safety contributing to a more harmonious and resilient social environment. It can thus be concluded that cultural confidence plays a significant role in enhancing psychological safety within rural communities.

Therefore, leads to the third hypothesis:

*H3*: Cultural confidence can enhance psychological safety among rural residents.

### 2.5 Sense of collective belonging and psychological sense of safety

The feeling of belonging to a cohesive and supportive group plays a fundamental role in shaping psychological safety. According to social support theory (Sarason, 2013), individuals who perceive their social networks as supportive are less likely to experience stress and more likely to maintain emotional resilience. In rural communities where collective activities, such as traditional music festivals, are common, these experiences contribute to the development of strong interpersonal connections. These collective experiences foster a sense of emotional and social security, creating a sense of belonging that acts as a buffer against psychological threats (Allen, 2020; Heard and Bartleet, 2025).

When rural residents feel that they belong to a supportive group, they perceive their environment as safe and welcoming. This sense of belonging creates a foundation for psychological safety, where individuals can express themselves openly without fear of rejection. Additionally, collective belonging enhances social solidarity, which ensures that individuals are supported emotionally and psychologically by their peers (Allen et al., 2021). The shared engagement in cultural practices such as music festivals strengthens this solidarity, reinforcing a collective sense of security (Lai et al., 2025).

Given the mechanisms described, it can be inferred that the sense of collective belonging in rural communities directly contributes to psychological safety. By feeling part of a supportive and cohesive group, individuals are better able to navigate social challenges and express themselves authentically without fear.

Therefore, leads to the fourth hypothesis:

*H4*: Sense of collective belonging among rural residents can enhance their psychological safety.

### 2.6 The moderating role of life satisfaction

Life satisfaction, a central dimension of subjective well-being, has been shown to significantly influence how individuals perceive and interact with their social environments (Swami et al., 2025). As a reflection of overall contentment with life, it plays a crucial role in shaping individuals’ emotional and psychological responses to external factors, including social interactions and cultural activities. When individuals report higher life satisfaction, they tend to exhibit greater resilience, positive emotional regulation, and a higher propensity to engage in social activities, all of which foster deeper interpersonal connections (Chia et al., 2025).

In the context of Hubei’s rural communities, life satisfaction enhances individuals’ ability to engage meaningfully in cultural events, such as traditional music festivals, by fostering a stable emotional foundation. Residents who are content with their lives are more likely to participate in such cultural practices, which serve as avenues for both personal and collective expression. Engaging in these events not only strengthens their connection to their cultural heritage but also reinforces their bonds with the community. This process is informed by social integration theory (Blau, 1960), which suggests that individuals with higher levels of emotional stability are more likely to experience a sense of social cohesion and collective identity.

Moreover, life satisfaction acts as an emotional resource that amplifies the positive effects of cultural confidence on social engagement. Self-determination theory (Blau, 1960) argues that individuals who are emotionally stable and satisfied with their lives are more likely to feel competent, autonomous, and connected in social contexts, thereby enhancing their ability to benefit from cultural validation. For rural residents in Hubei, life satisfaction facilitates a heightened openness to social interactions and the recognition of their cultural identity, thus enhancing their cultural confidence and fostering deeper engagement in community life.

In this sense, life satisfaction can be conceptualized as a moderator in the relationship between cultural confidence and collective belonging. When individuals are satisfied with their lives, they are better equipped to internalize and integrate the cultural affirmations provided by traditional music festivals. Consequently, the positive effects of cultural confidence on individuals’ sense of belonging are strengthened. In other words, life satisfaction enhances the capacity for cultural participation to create stronger, more meaningful social bonds, reinforcing the link between cultural confidence and collective belonging.

Therefore, leads to the following hypothesis:

*H5*: Life satisfaction positively moderates the effect of cultural confidence on collective belonging among rural residents.

In addition to moderating the effect of cultural confidence on collective belonging, life satisfaction also plays a key role in moderating the relationship between cultural confidence and psychological safety. Psychological safety refers to an individual’s perception that they can express themselves without fear of judgment, rejection, or negative consequences (Frazier et al., 2017). Life satisfaction contributes to psychological safety by providing emotional stability, which enables individuals to feel secure in their social interactions and cultural expressions. Self-affirmation theory (Howell, 2017) suggests that individuals with a high sense of well-being are less likely to experience anxiety in social contexts, as they are emotionally more resilient and confident in their self-worth. This emotional balance allows them to navigate social settings, such as cultural festivals, with greater ease and openness.

In rural Hubei, where traditional music plays a significant role in community life, individuals who are more satisfied with their lives are likely to feel greater psychological safety in expressing their cultural identity. Life satisfaction, as an emotional resource, strengthens the relationship between cultural confidence and psychological safety by providing a buffer against social anxieties and fostering a sense of belonging (Pai et al., 2024). This emotional security ensures that individuals feel more confident in their cultural expressions and more secure in their interactions within the community (Koehler and Neubauer, 2020).

Thus, life satisfaction not only enhances the impact of cultural confidence on collective belonging but also magnifies its positive effects on psychological safety. When life satisfaction is high, rural residents are more likely to experience a strong sense of security in their social interactions, contributing to their overall well-being and reinforcing the cohesion of the community.

Therefore, leads to the following hypothesis:

*H6*: Life satisfaction positively moderates the effect of cultural confidence on psychological safety among rural residents.

**Figure 2:**
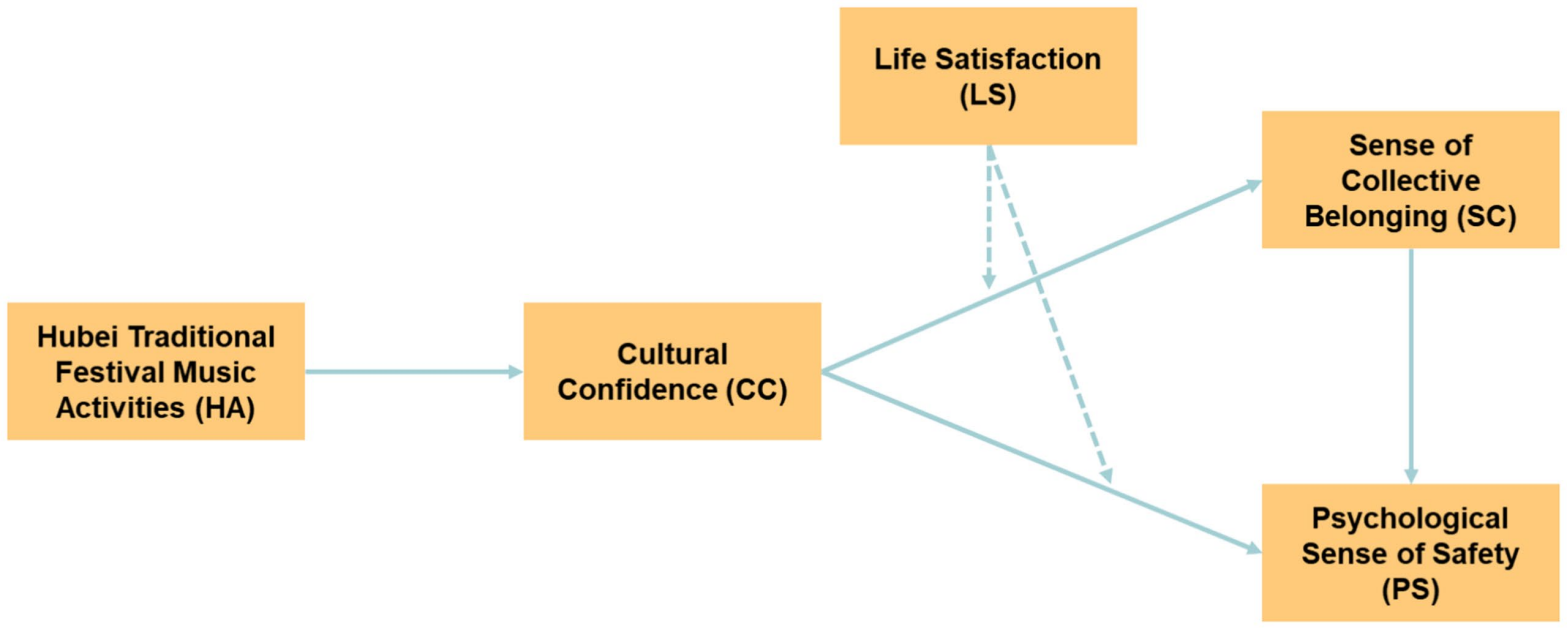
Research hypotheses and model of this study.

In conclusion, Figure 2 illustrates all the research hypotheses and the model utilized in this study.

## 3 Research methodology

### 3.1 Recruitment of respondents and data collection

The recruitment of participants was carried out with the collaboration and help of local village committees rural Hubei. These committees played a key role in identifying adult residents who had participated in at least one Hubei traditional festival music activity (HA) in the previous year. Participants were invited to volunteer for the study, and their involvement was purely voluntary. They were informed about the study’s objectives, and written consent was obtained.

To facilitate the recruitment process, a performance activity was organized by local cultural organizers, designed to showcase the unique musical elements of Hubei’s traditional festival music. This event aimed to not only draw participants into the study but also allow them to experience firsthand the musical characteristics that form the core of the research. The performance was held in a central community space, accessible to all rural residents.

The event featured traditional Hubei music, with distinct musical characteristics rooted in the region’s cultural heritage. Key features of the music included:

1. *Traditional instruments*: the performance showcased local instruments such as the erhu (a two-stringed bowed instrument), pipa (a pear-shaped plucked string instrument), dizi (a bamboo flute), and gong and drum ensembles. These instruments are emblematic of the region’s folk music traditions and are integral to local festival celebrations.
2. *Folk melodies and rhythms*: the music was based on regional folk melodies, with specific rhythms designed to evoke communal participation. These traditional tunes are often repetitive and rhythmic, allowing for group involvement such as collective singing or drumming.
3. *Choral singing and call-and-response patterns*: a common feature of Hubei’s traditional festival music is choral singing, with a prominent call-and-response structure that encourages community participation. This participatory nature aligns with the study’s focus on sense of collective belonging (SC), as the audience was invited to join in with simple refrains during the performance.
4. *Celebratory and ritualistic themes*: the music played during the festival is often associated with celebratory and ritualistic purposes, marking significant events such as harvest celebrations or local religious festivals. These performances often carry an emotional resonance that aligns with themes of cultural pride, heritage preservation, and community solidarity, which are key variables in this study.

After the performance, participants were invited to fill out the self-administered questionnaire, which measured their experiences with Hubei traditional festival music activities (HA), their levels of cultural confidence (CC), sense of collective belonging (SC), psychological sense of safety (PS), and life satisfaction (LS). The event provided a unique context for the respondents to reflect on their emotional and psychological responses to the traditional music, facilitating a deeper understanding of the impact of these activities on their well-being.

We employed a purposive sampling technique to recruit participants who had firsthand experience with Hubei traditional festival music activities. This approach ensures that our findings are grounded in the perspectives of individuals who can provide rich insights into the cultural significance and psychological impact of these activities.

To ensure participant eligibility, we used specific screening questions during the recruitment phase: “Have you participated in any Hubei traditional festival music activities in the past year?” Only those who met these criteria were invited to participate in the study.

### 3.2 Measuring tool

The measurement tools for this study were adapted from validated scales in existing literature on cultural participation, social psychology, and community well-being. These tools were modified to ensure their relevance and appropriateness for the rural Hubei context. The scales were pilot tested to assess their clarity and reliability, yielding high internal consistency (Cronbach’s alpha > 0.700) for all key constructs.

In this study, to accurately measure the impact of Hubei traditional festival music activities on the collective sense of belonging and psychological safety of rural residents, we designed three measurement items for each research variable. Each research variable was rated using a 5-point Likert scale, quantifying responses from “strongly disagree” to “strongly agree.” Table 1 presents the specific measurement items.

**Table 1:** Measurement items.

Research variables	Measurement items	Sources
Hubei traditional festival music activities (HA)	HA1: The melodic structure of Hubei traditional festival music has significant local characteristics, allowing me to experience a strong regional cultural atmosphere. HA 2: In Hubei traditional festival music activities, the use of percussion instruments (such as gongs and drums) and folk instruments (such as suona and erhu) enhance the festive atmosphere and emotional intensity of the events. HA 3: The rhythmic variations in Hubei traditional festival music activities are rich and expressive, capable of conveying strong emotional fluctuations and a festive atmosphere.	Mingfeng and Pattananon (2024) and Tang (2021)
Cultural confidence (CC)	CC1: I am proud of the uniqueness and richness of Hubei’s traditional festival music culture. CC2: I believe that Hubei traditional festival music represents the cultural essence of our nation. CC3: In my daily life, I actively introduce Hubei’s traditional festival music and culture to others.	Pan et al. (2021) and Yin et al. (2023)
Sense of collective belonging (SC)	SC1: Participating in Hubei traditional festival music activities makes me feel like a part of the community. SC2: I believe that through Hubei traditional festival music activities, my connection with community members has become stronger. SC3: In Hubei traditional festival music activities, I can feel the bond and shared cultural goals among community members.	Glasford (2021) and Esters et al. (2023)
Psychological sense of safety (PS)	PS1: I believe that Hubei traditional festival music activities provide an open and friendly atmosphere that allows people to participate with ease. PS2: When participating in Hubei traditional festival music activities, I do not worry about being ridiculed or excluded because of my opinions or ways of participation. PS3: While engaging in Hubei traditional festival music activities, I feel that the people around me respect and accept my way of participation and expression, which gives me a sense of security.	Aboramadan and Kundi (2023) and Hasan and Kashif (2021)
Life satisfaction (LS)	LS1: I feel satisfied with my current life. LS2: I believe my life is very close to what I envision as ideal. LS3: Overall, I think the state and quality of my life have reached a level that I find satisfactory.	Carlsson and Hamrin (2002) and Lättman et al. (2019)

Each of these tools was carefully adapted for the rural Hubei setting, and local cultural experts were consulted to ensure the scales accurately reflected the respondents’ experiences with the traditional music and the festival activities. The items were tested for clarity and relevance, with modifications made to ensure that the respondents fully understood the questions and that the scales were contextually appropriate.

In addition, in the process of developing and refining the measurement items of psychological sense of safety (PS), we drew upon the concept of psychological safety, which, although psychological safety has been most frequently examined in organizational and team-based settings, its core meaning—feeling free from negative social evaluation when engaging with others—is not context-bound to the workplace. In community-based cultural contexts such as rural traditional festival music activities, psychological safety manifests as residents’ perceived ability to participate, express themselves, and interact with others without fear of ridicule, exclusion, or disapproval. Such perceptions are particularly salient in rural collective rituals, where participation and expression are highly visible and socially embedded.

Finally, a series of reliability and validity tests were conducted. A pilot survey was conducted with 50 selected participants, including qualitative and quantitative internal consistency tests and construct validity assessments. In addition, face-to-face interviews were conducted to assess the validity of the initial scale and to revise the questionnaire. Key changes included rewording ambiguous items for clarity, adding locally relevant examples to enhance understanding, and removing questions that participants found confusing. This iterative process refined the questionnaire, ensuring that it was contextually appropriate and engaging for respondents.

In conclusion, this research design for this study was rigorously structured to ensure the validity and reliability of the findings.

### 3.3 Respondent information

A total of 429 participants were initially recruited for the study. After eligibility and data integrity checks, 378 valid questionnaires were retained for analysis, resulting in an effective response rate of 88.112%.

**Figure 3:**
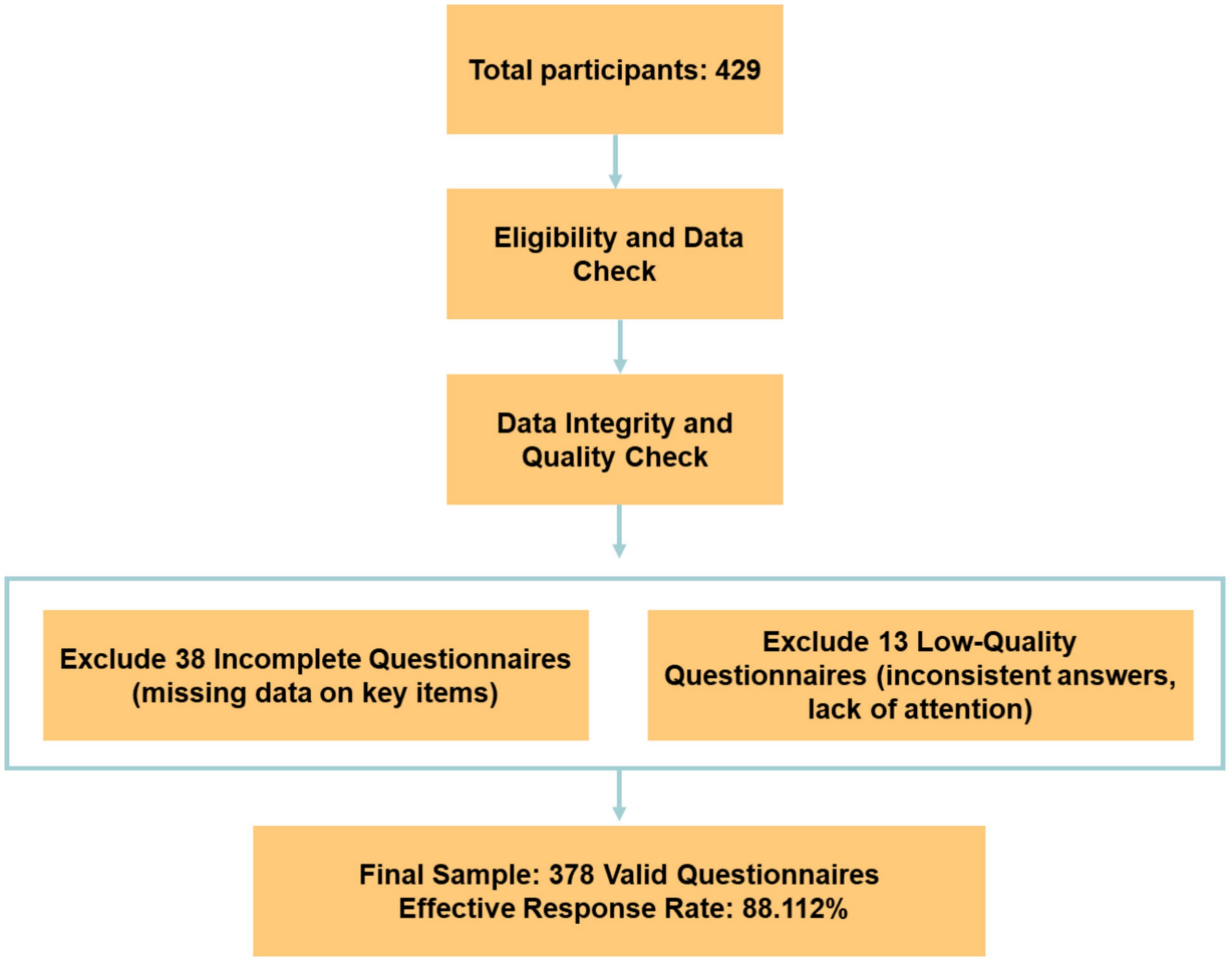
Participant flow.

More specifically (see Figure 3), after the initial data collection, 51 questionnaires were excluded from the analysis, the exclusion was based on the following criteria: (1) Incomplete Responses: 38 questionnaires were excluded due to missing data on key items, which compromised the ability to conduct reliable analysis. (2) Low-Quality Responses: 13 questionnaires were excluded due to poor-quality responses, such as inconsistent or contradictory answers, or responses that clearly indicated lack of attention or understanding during completion (e.g., selecting the same answer across all items, or nonsensical answers). All excluded questionnaires were reviewed to ensure that the criteria for exclusion were applied consistently and transparently.

Table 2 provides a detailed breakdown of the participants’ demographic information, including gender, age, education level, and other key characteristics.

**Table 2:** Information of 378 respondents.

Categories	Options	Frequency	Percentage
Gender	Male	185	48.9%
	Female	193	51.1%
Marriage	Singles and others	180	47.6%
	Married	198	52.4%
Age	18–24	34	9.0%
	25–34	70	18.5%
	35–44	74	19.6%
	45–54	73	19.3%
	55–64	74	19.6%
	>64	53	14.0%
Education level	Undergraduate and below	276	73.0%
	Postgraduate and above	102	27.0%
Average monthly income (CNY)	<4,000	80	21.2%
	4,000–5,000	82	21.7%
	5,000–6,000	85	22.5%
	6,000–7,000	77	20.4%
	>7,000	54	14.3%
Do you have any religious beliefs?	Yes	99	26.2%
	No	279	73.8%

### 3.4 Informed consent procedure

Prior to data collection, a standardized written informed consent procedure was implemented for all participants. Before completing the questionnaire, participants were provided with a consent form written in clear and accessible language, which explained the study’s objectives, the type of data to be collected, the expected time commitment, and the voluntary nature of participation. Participants were explicitly informed that they could refuse participation or withdraw at any point without any negative consequences. The consent form also stated that no personally identifiable information would be collected and that all responses would be used solely for academic research purposes. Only participants who confirmed their understanding and signed the consent form were included in the study. This procedure ensured transparency and participant autonomy while remaining proportionate to the minimal-risk nature of the research.

### 3.5 Anonymization and data access and control measures

To protect participants’ privacy, the study adopted a strict anonymization protocol at all stages of data collection and analysis. No names, identification numbers, contact information, or location-specific identifiers were collected. Each questionnaire was assigned a numerical code that could not be linked to an individual respondent. All data were stored in encrypted digital files, accessible only to the principal investigators and authorized members of the research team. Data analysis was conducted exclusively at the aggregate level, and no individual-level data were reported. Access to the raw dataset was restricted, and the data will not be shared with third parties. Upon completion of the research, the dataset will be securely archived or destroyed in accordance with institutional data management policies. These measures ensured that participant confidentiality and data security were fully maintained.

### 3.6 Ethical treatment

From a methodological perspective, this study was designed as a cross-sectional, non-interventional social survey, employing standardized self-report questionnaires to collect attitudinal and perceptual data. The research did not involve experimental manipulation, behavioral intervention, deception, or exposure to risk-inducing stimuli. All variables measured reflected participants’ routine cultural experiences and subjective perceptions (e.g., collective belonging and psychological safety) within their everyday social environment. In accordance with widely accepted methodological standards in social science research, such survey-based designs are classified as minimal-risk research, as they do not alter participants’ behavior, health status, or social conditions.

The use of written informed consent in this study reflects adherence to core ethical principles—namely respect for autonomy and voluntary participation. Methodologically, informed consent serves as a procedural safeguard to ensure transparency, participant comprehension, and voluntary engagement, even in studies that are exempt from formal ethical review. In social science research, particularly in field-based surveys involving community populations, obtaining written consent is considered best practice for data integrity and participant protection.

### 3.7 Structural equation modeling (SEM) method

The choice to employ structural equation modeling (SEM) in this study was driven by the complexity of the hypothesized relationships between the variables and the need for a comprehensive analytical approach. SEM is particularly suitable for research that seeks to understand the interrelationships between multiple latent constructs [such as cultural confidence (CC), sense of collective belonging (SC), and psychological sense of safety (PS)] and their direct or indirect effects on one another. In this study, the relationships between Hubei traditional festival music activities (HA), cultural confidence (CC), sense of collective belonging (SC), psychological sense of safety (PS) and life satisfaction (LS) are not only theoretically complex but also require the ability to model both direct and mediating effects. SEM allows for the simultaneous analysis of multiple dependent and independent variables, providing a more nuanced understanding of the paths and interactions between them, as opposed to traditional regression techniques.

Moreover, SEM is widely regarded as a robust tool in the social sciences for testing theoretical models. It is particularly useful in research areas involving psychosocial constructs and latent variables, which are not directly observable but are inferred through multiple observed indicators, such as survey items (Hidayat and Wulandari, 2022). In this study, latent constructs like cultural confidence (CC) and sense of collective belonging (SC) could not be directly measured, making SEM the ideal choice for modeling their relationships with other variables. The method’s ability to handle complex models involving measurement error and model fit indices further supports its application (Owolabi et al., 2020). Additionally, SEM’s capacity to test mediating and moderating effects, as demonstrated in this study’s analysis of the role of life satisfaction (LS), makes it a particularly powerful tool for examining how psychological constructs interact in real-world settings (Dragan and Topolšek, 2014).

## 4 Empirical test

### 4.1 Assessment of measurement model

First, the fit indices for the model reveal a chi-square to degrees of freedom ratio of 1.039 accompanied by a CFI of 0.998, an RMSEA of 0.010, a TLI of 0.998, GFI of 0.977 and an IFI of 0.998. These results suggest that the model demonstrates an acceptable fit (West et al., 2012).

Next, to assess the existence of common method bias, the one-factor test suggested by Harman was employed. The findings revealed that the initial unrotated factor represented 28.003% of the variance, falling short of the 30% benchmark (Aguirre-Urreta and Hu, 2019). Then, further testing was conducted using the CFA comparison method (Bozionelos and Simmering, 2022), which involved comparing the original model with the model constructed from all item indicators. The results indicated a change in Δ*χ*^2^ of 1132.677, with ΔDf of 10 and a result of *p* < 0.05, meeting the criteria for excluding CMB. Consequently, it can be concluded that common method bias risk appears low.

Table 3 portrays the results concerning the reliability of the measurement tools along with their respective validity evaluations. Farrell (2010) emphasizes the importance of achieving an average variance extracted (AVE) score of no less than 0.5 to establish validity. Furthermore, this research utilized Cronbach’s alpha coefficient paired with composite reliability metrics to assess the validity of the survey instrument. A coefficient exceeding 0.7 suggests that the survey is considered reliable (Safiih and Azreen, 2016). The data illustrated in the tables indicate that the internal consistency measures for all instruments employed in this research are regarded as acceptable.

**Table 3:** Reliability and validity test.

Variables	Items	Standardized coefficients	Standard error of the mean (SE)	*Z*-value	*p*	Average variance extracted (AVE)	Composite reliability (CR)	Cronbach’s alpha
Hubei traditional festival music activities (HA)	HA1	0.748				0.542	0.780	0.779
	HA2	0.713	0.083	11.206	***			
	HA3	0.747	0.079	11.364	***			
Cultural confidence (CC)	CC1	0.728				0.586	0.809	0.808
	CC2	0.786	0.084	12.797	***			
	CC3	0.782	0.085	12.771	***			
Sense of collective belonging (SC)	SC3	0.753				0.542	0.780	0.777
	SC2	0.712	0.083	11.479	***			
	SC1	0.742	0.074	11.695	***			
Psychological sense of safety (PS)	PS3	0.837				0.788	0.918	0.915
	PS2	0.883	0.050	21.801	***			
	PS1	0.940	0.046	23.504	***			
Life satisfaction (LS)	LS1	0.830				0.621	0.831	0.829
	LS2	0.799	0.063	14.753	***			
	LS3	0.732	0.059	13.882	***			

***Represents significance with a *p*-value less than 0.01.

According to Cheung and Wang (2017), the confirmation of discriminant validity occurs when the AVE for each construct is greater than the shared variance present among other constructs. This can be illustrated by the square root of the AVE being greater than the correlation coefficients, as illustrated in Table 4, signifying that the measurement items exhibit robust discriminant validity (Farrell and Rudd, 2009).

**Table 4:** Discriminant validity test.

Variables	Life satisfaction (LS)	Psychological sense of safety (PS)	Sense of collective belonging (SC)	Cultural confidence (CC)	Hubei traditional festival music activities (HA)
Life satisfaction (LS)	0.788				
Psychological sense of safety (PS)	0.466	0.888			
Sense of collective belonging (SC)	0.281	0.492	0.736		
Cultural confidence (CC)	0.190	0.452	0.146	0.766	
Hubei traditional festival music activities (HA)	0.173	0.290	0.205	0.330	0.736

Table 5 displays the results obtained from the exploratory factor analysis. Each measurement variable demonstrated factor loadings greater than 0.600 (Hooper, 2012). Consequently, the results of the exploratory factor analysis are considered robust.

**Table 5:** Exploratory factor analysis.

Items		Factors				
		1	2	3	4	5
Psychological sense of safety (PS)	PS3	**0.861**	0.213	0.205	0.225	0.095
	PS2	**0.858**	0.195	0.196	0.182	0.092
	PS1	**0.848**	0.171	0.162	0.192	0.116
Life satisfaction (LS)	LS1	0.179	**0.858**	−0.039	0.117	0.043
	LS2	0.134	**0.847**	0.101	0.104	0.026
	LS3	0.158	**0.818**	0.103	0.023	0.082
Cultural confidence (CC)	CC1	0.192	0.054	**0.785**	0.014	0.147
	CC2	0.109	0.090	**0.856**	0.009	0.082
	CC3	0.153	0.021	**0.837**	0.067	0.094
Sense of collective belonging (SC)	SC3	0.107	0.112	0.104	**0.826**	0.048
	SC2	0.200	0.099	−0.004	**0.784**	0.025
	SC1	0.166	0.023	−0.013	**0.823**	0.093
Hubei traditional festival music activities (HA)	HA1	0.169	0.068	0.091	0.031	**0.810**
	HA2	0.037	0.063	0.106	0.037	**0.817**
	HA3	0.040	0.013	0.107	0.095	**0.831**

The bolded numbers represent factor loads.

### 4.2 Assessment of structural model

In Table 6, the research hypotheses are displayed, and the findings confirm that hypotheses 1 through 4 are supported, demonstrating significant positive relationships across these pathways of influence. This conclusion is consistent with academic criteria, as the z-value surpasses 1.96 and the *p*-value is deemed statistically significant (Kite and Whitley, 2018).

**Table 6:** Research hypothesis test - direct effect.

Research Hypothesis	Paths			S.E.	Z	Std.	*p*	Result
H1	Hubei traditional festival music activities (HA)	→	Cultural confidence (CC)	0.064	5.138	0.345	***	Established
H2	Cultural confidence (CC)	→	Sense of collective belonging (SC)	0.070	2.409	0.157	**	Established
H3	Cultural confidence (CC)	→	Psychological sense of safety (PS)	0.060	7.001	0.395	***	Established
H4	Sense of collective belonging (SC)	→	Psychological sense of safety (PS)	0.058	7.365	0.430	***	Established

***Represents significance with a *p*-value less than 0.01; **represents significance with a *p*-value less than 0.05.

The moderating effect was evaluated utilizing the PROCESS 4.0 plugin within SPSS, with findings outlined in Table 7. The analysis indicated that when the dependent variable is the sense of collective belonging (SC), the interaction effect observed between cultural confidence (CC) and life satisfaction (LS) is 0.108, accompanied by a p-value lower than 0.05. Furthermore, the 95% confidence interval does not encompass 0 (LLCI: 0.124, ULCI: 0.248). Such evidence implies that life satisfaction (LS) serves a positive moderating function in the relationship between cultural confidence (CC) and sense of collective belonging (SC), thereby providing support for research hypothesis 5.

**Table 7:** Moderation effects test.

Dependent Variable	Independent variable	Unstd	SE	*T*	*p*	LLCI	ULCI
Sense of collective belonging (SC)	Cultural confidence (CC)	0.177	0.050	3.138	***	0.101	0.275
	Life satisfaction (LS)	0.197	0.048	4.117	***	0.103	0.292
	Cultural confidence (CC) × life satisfaction (LS)	0.168	0.059	2.336	***	0.124	0.248
Psychological sense of safety (PS)	Cultural confidence (CC)	0.341	0.045	7.500	***	0.251	0.430
	Life satisfaction (LS)	0.347	0.044	7.949	***	0.261	0.432
	Cultural confidence (CC) × life satisfaction (LS)	0.221	0.054	3.516	***	0.155	0.306

***Represents significance with a *p*-value less than 0.01.

In addition, when considering psychological sense of safety (PS) as the dependent variable, the interaction between cultural confidence (CC) and life satisfaction (LS) yields a value of 0.221, accompanied by a *p*-value below 0.05. Moreover, the 95% confidence interval does not encompass 0 (LLCI: 0.155, ULCI: 0.306). This suggests that life satisfaction (LS) functions as a positive moderator in the relationship between cultural confidence (CC) and psychological sense of safety (PS), thus supporting research hypothesis 6.

## 5 Summary and discussion

### 5.1 Theoretical contributions

This study offers several key theoretical contributions that deepen our understanding of the role of traditional music activities in shaping cultural identity, collective belonging, and psychological safety in rural Hubei. It advances the theoretical discourse by highlighting the psychological and social mechanisms through which cultural confidence, facilitated by traditional festival music, influences community dynamics in rural contexts.

One of the primary contributions of this research is its expansion of the theory of cultural identity through the lens of music. While existing literature has underscored the importance of cultural practices in the construction of identity, this study emphasizes the central role of traditional festival music as a dynamic platform for reinforcing cultural self-concept. By focusing specifically on rural Hubei, the research adds a new dimension to the understanding of how music serves as a powerful tool for emotional and psychological engagement with one’s cultural heritage. Through participation in traditional music activities, individuals reconnect with their community’s history and cultural roots, reinforcing their sense of self and group membership. This contribution broadens the existing theoretical framework of cultural identity (Li and Wood, 2016; Tao et al., 2020) by offering empirical evidence of how traditional music can deepen cultural connections and fortify individual and collective identities. This study further enriches the theoretical understanding of cultural identity by introducing a rural, music-centered perspective that has not been extensively addressed in previous studies.

Additionally, this study integrates social identity theory with cultural confidence, offering a nuanced understanding of the role of cultural practices in group cohesion. Drawing on the works of Ellemers and Haslam (2012) and Bohlman (1988), it demonstrates how cultural markers, such as traditional music, play a central role in forming and maintaining social bonds. In rural Hubei, these music activities are not just expressions of cultural pride, but essential mechanisms for individuals to affirm their group identity. The research shows that as individuals engage with cultural practices, they internalize and validate the shared values of their community, which in turn strengthens their emotional attachment to the group. This connection between cultural confidence and group identity adds new dimensions to social identity theory by illustrating how cultural engagement does not only enhance individual self-concept but also reinforces the social fabric of the community.

This study also offers valuable insights into the psychological mechanisms that underlie the formation of collective belonging. By demonstrating how cultural confidence contributes to a deeper emotional investment in the community, it extends the work of Scheepers and Ellemers (2019) and others in social identity research. The research reveals that when individuals feel confident in their cultural background, they are more likely to strengthen their ties to the community, viewing the group as integral to their sense of self. The study also shows that the celebration of cultural traditions, particularly through shared musical experiences, creates a sense of collective purpose, reinforcing the bonds between individuals. This contribution enriches our understanding of how cultural confidence serves as the foundation for social cohesion and collective identity in rural settings.

Furthermore, this research makes a significant contribution by linking cultural confidence with psychological safety, an area that has not been fully explored in existing studies on cultural practices and social well-being. Psychological safety, defined as the feeling that one’s identity is accepted and valued within a group, is shown to be closely tied to cultural confidence in rural Hubei. This contribution is particularly important in highlighting how cultural practices, such as traditional music festivals, can provide emotional security and foster a sense of safety. The connection between cultural confidence and psychological safety not only expands our understanding of how cultural activities influence individual well-being but also adds depth to existing theories of social inclusion and self-affirmation.

Another notable theoretical contribution of this study is its exploration of the moderating role of life satisfaction in the relationships between cultural confidence, collective belonging, and psychological safety. While life satisfaction has been widely studied in relation to individual well-being, its role as a moderator in the context of cultural practices is underexplored. This study sheds light on how individuals with higher life satisfaction are more likely to participate in and benefit from cultural activities, such as traditional music festivals, by fostering greater emotional resilience and social integration. By conceptualizing life satisfaction as a moderator, the research offers new insights into how emotional stability enhances the positive effects of cultural confidence on social engagement and collective identity. This finding adds a new layer to theories of social participation and cultural validation by showing how well-being serves as a crucial emotional resource that amplifies the benefits of cultural involvement.

Moreover, this study advances the concept of cultural confidence itself, suggesting that it is not just an individual trait but a socially constructed phenomenon that is shaped and reinforced through collective cultural practices. In rural Hubei, cultural confidence is nurtured through participation in traditional music events, which serve as vital spaces for affirming and celebrating cultural heritage. This perspective broadens our understanding of cultural confidence, showing that it is a dynamic and relational concept that thrives within the social context of community engagement. By framing cultural confidence in this way, the study extends existing conceptualizations and offers a more comprehensive view of how cultural practices contribute to both individual and collective psychological well-being.

Also, this research paves the way for future studies to explore the role of traditional cultural practices in fostering community resilience and psychological well-being, particularly in rural areas where cultural heritage is at risk of being overshadowed by modernization. By providing empirical evidence of the positive psychological and social outcomes associated with cultural engagement, the study encourages further exploration into how these practices can be leveraged for community development and cultural sustainability. The integration of life satisfaction as a moderating factor also opens new avenues for investigating how individual well-being interacts with collective cultural participation to foster stronger, more resilient communities.

Beyond reinforcing existing frameworks, this study directly addresses a notable gap in the literature by integrating the concepts of cultural confidence, collective belonging, and psychological safety into a unified explanatory model grounded in the socio-cultural reality of rural communities. While prior research on cultural identity and social cohesion (e.g., Newman et al., 2017; Roussin et al., 2016) has tended to treat psychological safety as either an organizational factor or an implicit outcome of group affiliation, our findings explicitly position it as a culturally mediated construct. This theoretical integration extends the boundaries of social identity theory by demonstrating that cultural practices—specifically traditional festival music—do not simply reflect existing group identities but actively construct an environment that safeguards those identities, thereby fostering emotional security. As such, the research links micro-level emotional processes to macro-level community cohesion, offering a nuanced understanding that bridges cultural psychology with rural sociology.

Finally, this research makes an original theoretical contribution by providing a contextualized expansion of musicology’s role in social theory. Traditional ethnomusicological studies have largely documented the preservation of heritage through communal music-making but have seldom explicated the mechanisms by which music shapes psychological safety and collective belonging in geographically and socially specific communities. By empirically demonstrating how rural Hubei’s festival music strengthens cultural confidence and simultaneously moderates the impacts of modernization and social fragmentation, our work advances existing literature from descriptive accounts toward a predictive, theory-driven framework. This shift enables scholars to better theorize the protective role of intangible cultural heritage in social resilience, positioning music not merely as symbolic heritage but as an active agent in sustaining collective identity and psychological well-being.

In conclusion, this study makes several significant theoretical contributions that advance our understanding of the relationship between cultural practices, community cohesion, and psychological well-being. By linking cultural confidence with collective belonging and psychological safety, and by introducing life satisfaction as a moderating factor, the research offers fresh insights into the complex interplay of cultural identity, social engagement, and emotional security in rural communities. These contributions not only extend existing theories in cultural studies and social psychology but also offer practical implications for enhancing community well-being through cultural practices.

### 5.2 Practical and managerial contributions

The empirical results of this study show that Hubei traditional festival music activities play a significant role in enhancing rural residents’ cultural confidence, which further promotes a stronger sense of collective belonging and a higher psychological sense of safety. Moreover, life satisfaction strengthens the positive effects of cultural confidence on both sense of collective belonging and psychological sense of safety. These findings not only confirm the cultural and psychological value of Hubei traditional festival music activities but also highlight its potential as a practical tool for local governments, cultural managers, and rural development planners. Building upon these conclusions, it is possible to outline a series of actionable recommendations to integrate Hubei traditional festival music activities into rural revitalization strategies, community governance, and social well-being programs.

Local governments and cultural management authorities should integrate traditional festival music activities into long-term rural cultural development plans, ensuring that these events become stable and recurring components of community life. Implementation can include creating structured annual festival calendars, providing training programs for local musicians, and offering logistical and financial support to encourage broad participation. Festival events should be designed to actively engage residents through interactive performances, collective singing, and shared rituals, fostering deeper emotional connections and cognitive resonance among participants.

Festival organizers are encouraged to adopt participation models that embrace inclusivity across age, gender, and socioeconomic backgrounds. Multi-stage festival programs that invite residents to perform, assist in event preparation, and contribute culturally relevant musical content can enhance a sense of ownership and strengthen feelings of belonging. Promoting inter-village musical exchanges will further broaden collective identity beyond individual communities, solidifying bonds that contribute to a stronger collective belonging and improved psychological safety across rural areas.

Careful curation of festival activities can enhance cultural confidence, which supports psychological safety. This involves highlighting local musical heritage, weaving narratives of cultural pride into performances, and recognizing the contributions of local musicians. Creating physically comfortable, socially welcoming, and emotionally resonant environments enables participants to experience lower levels of anxiety and greater feelings of safety. Pre- and post-event surveys can be used to assess shifts in psychological safety and inform adjustments to future event designs for greater impact.

The moderating role of life satisfaction suggests that combining cultural initiatives with broader improvements in quality of life can amplify the benefits of Hubei traditional festival music activities. Policymakers should seek coordinated action between cultural bureaus, social services, and economic development offices to enhance public services, recreational facilities, and economic opportunities in rural communities. Linking festival participation with an improved living environment will create an integrated “culture-well-being” system in which cultural confidence, collective belonging, and psychological safety are mutually reinforced.

In sum, Hubei traditional festival music activities should be regarded as strategic instruments for strengthening cultural confidence, fostering collective belonging, and enhancing psychological safety in rural areas. Through thoughtful integration into rural governance, inclusive participation practices, carefully crafted event experiences, and alignment with initiatives that raise life satisfaction, local authorities can transform cultural traditions into sustainable drivers of community cohesion and long-term psychosocial well-being. These measures translate the empirical findings of this research into clear pathways for effective rural revitalization policy and practice.

### 5.3 Limitations and future studies

This study has several limitations that need to be explained, and should be considered for future research. First, this study is limited by its sample size and sampling scope. The data were exclusively drawn from 378 rural residents in Hubei, meaning that the findings may not be generalizable to other regions of China or internationally. Rural communities in other provinces may exhibit different cultural practices, social dynamics, and engagement with music, which could lead to varying effects of traditional festival music activities on psychological and social outcomes. Therefore, the generalizability of the results is confined to the rural areas of Hubei province, and further research could expand the sample to include other rural or urban populations across China or even other countries to test the robustness of these findings in different cultural contexts.

Second, this study exclusively focuses on the effects of Hubei traditional festival music activities (HA), which limits the scope of the research. While this focus provides valuable insights into the specific role of these cultural practices in rural community development, the study does not examine the effects of other types of cultural or musical activities, such as contemporary music festivals or other regional traditions. This could be a potential avenue for future research, as comparing different types of cultural engagement could reveal whether traditional music is uniquely effective in enhancing cultural confidence (CC), sense of collective belonging (SC), psychological sense of safety (PS) and life satisfaction (LS), or whether similar effects could be observed in other cultural contexts.

A third limitation lies in the cross-sectional design of the study. The research design focuses on a snapshot of data collected at a single point in time, which limits the ability to draw conclusions about causality. While the findings suggest strong relationships between HA, CC, SC, PS, and LS, a longitudinal study would provide more insights into the temporal dynamics of these relationships and could explore whether the effects of HA on CC, SC, and PS are sustained over time or whether they diminish as individuals experience different life events. Future research with a longitudinal approach would enable a more nuanced understanding of the long-term impact of traditional music activities on rural residents’ psychological well-being.

Additionally, the study is focused on a limited set of variables, namely CC, SC, PS, and LS. While these variables are highly relevant to the research questions, other psychological, social, and economic factors could play a significant role in shaping the outcomes. For instance, socioeconomic status, education level, and family structure might interact with the effects of HA, potentially influencing the development of CC, SC, and PS. Future studies could explore the influence of additional variables, such as social capital, economic development, or migration patterns, to offer a more comprehensive understanding of the multifaceted impacts of traditional music activities on rural communities.

Final, this study does not address the potential influence of individual differences in the response to HA. It assumes a general effect across the rural population, but individual factors such as age, gender, or prior exposure to traditional music could influence the outcomes. For example, younger generations may engage with traditional music in different ways compared to older generations, and this variation might lead to differences in CC and SC. Future research could investigate how individual differences modulate the effects of cultural participation, offering more personalized recommendations for cultural policy and rural development.

## Data Availability

The raw data supporting the conclusions of this article will be made available by the authors, without undue reservation.

## References

[ref1] Aboramadan M. Kundi Y. M. (2023). Emotional culture of joy and happiness at work as a facet of wellbeing: a mediation of psychological safety and relational attachment. Pers. Rev. 52, 2133–2152. doi: 10.1108/PR-04-2021-0285

[ref2] Aguirre-Urreta M. I. Hu J. (2019). Detecting common method bias: performance of the Harman’s single-factor test. Data Base 50, 45–70. doi: 10.1145/3330472.3330477

[ref3] Allen K. A. (2020). The psychology of belonging. Abingdon: Routledge.

[ref4] Allen K. A. Kern M. L. Rozek C. S. McInerney D. M. Slavich G. M. (2021). Belonging: a review of conceptual issues, an integrative framework, and directions for future research. Aust. J. Psychol. 73, 87–102. doi: 10.1080/00049530.2021.1883409, 33958811 PMC8095671

[ref5] Bartleet B. L. (2016). “‘Pride in self, Pride in community, Pride in culture’: the role of stylin’up in fostering indigenous community and identity” in The Festivalization of culture (Abingdon: Routledge).

[ref6] Blau P. M. (1960). A theory of social integration. Am. J. Sociol. 65, 545–556. doi: 10.1086/222785

[ref7] Bohlman P. V. (1988). Traditional music and cultural identity: persistent paradigm in the history of ethnomusicology. Yearb. Tradit. Music 20, 26–42. doi: 10.2307/768164

[ref8] Bozionelos N. Simmering M. J. (2022). Methodological threat or myth? Evaluating the current state of evidence on common method variance in human resource management research. Hum. Resour. Manag. J. 32, 194–215. doi: 10.1111/1748-8583.12398

[ref9] Carlsson M. Hamrin E. (2002). Evaluation of the life satisfaction questionnaire (LSQ) using structural equation modelling (SEM). Qual. Life Res. 11, 415–426. doi: 10.1023/a:1015670628990 12113389

[ref10] Carter M. J. Fuller C. (2015). Symbolic interactionism. Sociopedia. isa 1, 1–17. doi: 10.1177/205684601561

[ref11] Chalton R. A. McQuaid G. A. Wallace G. L. (2023). Social support and links to quality of life among middle-aged and older autistic adults. Autism 27, 92–104. doi: 10.1177/13623613221081917 35362329 PMC9806477

[ref12] Chen M. Zhou Y. Huang X. Ye C. (2021). The integration of new-type urbanization and rural revitalization strategies in China: origin, reality and future trends. Land 10:207. doi: 10.3390/land10020207

[ref13] Cheung G. W. Wang C. (2017). Current approaches for assessing convergent and discriminant validity with SEM: issues and solutions. In academy of management proceedings. Briarcliff Manor, NY: Academy of Management.

[ref14] Chia J. L. Hartanto A. Tov W. (2025). Supporting satisfaction, satisfying support: bidirectional associations of social support and life satisfaction. Soc. Psychol. Personal. Sci. 16, 758–768. doi: 10.1177/19485506241283584

[ref15] Chiya A. (2024). The role of rural music festivals in community resilience, resourcefulness, and well-being. Int. J. Soc. Sustain. Econ. Soc. Cultural Context 21:25. doi: 10.18848/2325-1115/cgp/v21i01/25-50

[ref16] De Leeuw R. N. Janicke-Bowles S. H. Ji Q. (2022). How music awakens the heart: an experimental study on music, emotions, and connectedness. Mass Commun. Soc. 25, 626–648. doi: 10.1080/15205436.2021.1956542

[ref17] De Witte M. Orkibi H. Zarate R. Karkou V. Sajnani N. Malhotra B. . (2021). From therapeutic factors to mechanisms of change in the creative arts therapies: a scoping review. Front. Psychol. 12:678397. doi: 10.3389/fpsyg.2021.678397, 34366998 PMC8336579

[ref18] de-Miguel-Molina B. Santamarina-Campos V. de-Miguel-Molina M. Boix-Doménech R. (2021). Music as intangible cultural heritage: economic, cultural and social identity. Berlin: Springer Nature.

[ref19] Dragan D. Topolšek D. 2014 “Introduction to structural equation modeling: review, methodology and practical applications.” In: The international conference on Logistics & Sustainable Transport

[ref20] Duffy M. (2018). Music events and festivals: Identity and experience the Routledge handbook of festivals. Abingdon: Routledge.

[ref21] Eaude T. (2020). Identity, culture and belonging. London: Bloomsbury Publishing.

[ref22] Ellemers N. Haslam S. A. (2012). “Social identity theory.” In: Lange P. A. M. Van Kruglanski A. W. Higgins E. T. (Eds.) Handbook of theories of social psychology Thousand Oaks, CA: Sage Publications.

[ref23] Esters P. Godor B. P. Van der Hallen R. (2023). Investigating the role of residential migration history on the relationship between attachment and sense of belonging: a SEM approach. J. Community Psychol. 51, 468–485. doi: 10.1002/jcop.22918, 35852147 PMC10084290

[ref24] Farrell A. M. (2010). Insufficient discriminant validity: a comment on Bove, Pervan, Beatty, and Shiu (2009). J. Bus. Res. 63, 324–327. doi: 10.1016/j.jbusres.2009.05.003

[ref25] Farrell A. M. Rudd J. M. 2009 “Factor analysis and discriminant validity: a brief review of some practical issues.” In: Australia and New Zealand marketing academy conference 2009. Sydney: ANZMAC

[ref26] Feiyan W. Punvaratorn M. Rodsakan T. (2024). Identity and musical culture construction of Xianning people in Hubei Province. J. Multidiscipl. Hum. Soc. Sci. 7, 2988–3008.

[ref27] Frazier M. L. Fainshmidt S. Klinger R. L. Pezeshkan A. Vracheva V. (2017). Psychological safety: a meta-analytic review and extension. Pers. Psychol. 70, 113–165. doi: 10.1111/peps.12183

[ref28] Gao Q. Shi S. (2025). Preserving cultural identity: strategies for integrating national musical heritage into global music platforms. Int. J. Inclusive Educ., 1–33. doi: 10.1080/13603116.2025.2556311

[ref29] Glasford D. E. (2021). Composition of place, minority vs. majority group-status, & contextualized experience: the role of level of group representation, perceiving place in group-based terms, and sense of belonging in shaping collective behavior. PLoS One 16:e0253571. doi: 10.1371/journal.pone.0253571, 34543265 PMC8452021

[ref30] Gouk P. (Ed.) (2017). Musical healing in cultural contexts. Abingdon: Routledge.

[ref31] Hallam S. Himonides E. (2022). The power of music: an exploration of the evidence. Cambridge: Open Book Publishers.

[ref32] Hasan F. Kashif M. (2021). Psychological safety, meaningfulness and empowerment as predictors of employee well-being: a mediating role of promotive voice. Asia Pac. J. Bus. Adm. 13, 40–59. doi: 10.1108/APJBA-11-2019-0236

[ref33] Heard E. Bartleet B. L. (2025). How can community music shape individual and collective well-being? A case study of a place-based initiative. Health Promot. J. Austr. 36:e921. doi: 10.1002/hpja.921, 39257211 PMC11806369

[ref34] Hidayat R. Wulandari P. (2022). Structural equation modelling (SEM) in research: narrative literature review. Open Access Indo. J. Soc. Sci. 5, 852–858. doi: 10.37275/oaijss.v5i6.141

[ref35] Hooper D. (2012). “Exploratory factor analysis” in Approaches to quantitative research – theory and its practical application: a guide to dissertation students. ed. Chen H. (Cork: Oak Tree Press).

[ref36] Howell A. J. (2017). Self-affirmation theory and the science of well-being. J. Happiness Stud. 18, 293–311. doi: 10.1007/s10902-016-9713-5

[ref37] Hu Q. Yang P. Ma J. Wang M. He X. (2024). The spatial differentiation characteristics and influencing mechanisms of intangible cultural heritage in China. Heliyon 10. doi: 10.1016/j.heliyon.2024.e38689, 39641020 PMC11617923

[ref38] Jian S. Chuangprakhon S. Santaveesuk P. (2024). Dongjing Chinese folk music in enhancing musical literacy and education. Int. J. Educ. Literacy Stud. 12, 151–158. doi: 10.7575/aiac.ijels.v.12n.4p.151

[ref39] Jiang Q. Qian J. Zang Y. (2026). Integrating intangible cultural heritage elements into mobile games: an exploration of player cultural identity. Inf. Technol. People 39, 482–500. doi: 10.1108/ITP-01-2024-0088

[ref40] Jiayang Li D. F. A. Su Y. (2024). Exploring the significance of traditional music in safeguarding and transmitting intangible cultural heritage: a case study of the Yunnan bai ethnic group. Cultura 21, 115–144.

[ref41] Jin X. B. Ye C. Yue W. Z. Ma L. B. Luo Z. D. Yang R. . (2024). Urban-rural integrated development in China in the new era: challenges and paths. J. Nat. Resour. 39, 1–28. doi: 10.31497/zrzyxb.20240101

[ref42] Kelmendi A. (2024). Sound identity as a phenomenon. Research on the cultural significance of music in ethnic and subcultural communities. Interdiscip. Cult. Humanit. Rev. 3, 16–23. doi: 10.59214/cultural/2.2024.16

[ref43] Kite M. E. Whitley B. E. (2018). “Factor analysis, path analysis, and structural equation modeling” in Principles of research in behavioral science (Abingdon: Routledge).

[ref44] Koehler F. Neubauer A. B. (2020). From music making to affective well-being in everyday life: the mediating role of need satisfaction. Psychol. Aesthet. Creat. Arts 14:493. doi: 10.1037/aca0000261

[ref45] Lai H. I. C. Thompson G. McFerran K. S. (2025). Music therapists’ perceptions of creating safety in the context of trauma with children and adolescents: a qualitative study. Nord. J. Music. Ther. 34, 174–194. doi: 10.1080/08098131.2024.2389552

[ref46] Laing J. Mair J. (2015). Music festivals and social inclusion–the festival organizers’ perspective. Leis. Sci. 37, 252–268. doi: 10.1080/01490400.2014.991009

[ref47] Lättman K. Olsson L. E. Friman M. Fujii S. (2019). Perceived accessibility, satisfaction with daily travel, and life satisfaction among the elderly. Int. J. Environ. Res. Public Health 16:4498. doi: 10.3390/ijerph16224498, 31739648 PMC6888452

[ref49] Li S. Li S. Fong L. H. N. Li Y. (2025). When intangible cultural heritage meets modernization–can Chinese opera with modernized elements attract young festival-goers? Tour. Manag. 107:105036. doi: 10.1016/j.tourman.2024.105036

[ref50] Li Y. N. Wood E. H. (2016). Music festival motivation in China: free the mind. Leis. Stud. 35, 332–351. doi: 10.1080/02614367.2014.962588

[ref51] Liebenberg L. Wall D. Wood M. Hutt-MacLeod D. (2019). Spaces & places: understanding sense of belonging and cultural engagement among indigenous youth. Int J Qual Methods 18:1609406919840547. doi: 10.1177/1609406919840547

[ref53] Liu Y. Song Y. (2025). The role of Chinese folk ritual music in biodiversity conservation: an ethnobiological perspective from the Lingnan region. J. Ethnobiol. Ethnomed. 21:6. doi: 10.1186/s13002-025-00755-7, 39881337 PMC11780889

[ref54] Mazlan C. A. N. Abdullah M. H. Nor Hashim N. S. Abdul Wahid N. (2025). Music in cultural tourism: insights from a dual approach of scoping review and bibliometric analysis. Humanit. Soc. Sci. Commun. 12, 1–17. doi: 10.1057/s41599-025-04847-3

[ref56] Mingfeng L. Pattananon N. (2024). Hubei folk song teaching methods in university in China. J. Mod. Learn. Dev. 9, 863–872.

[ref57] Modood T. Parekh B. Tyler C. Uberoi V. Connelly J. (2025). Multicultural conversations: the nature and future of culture, identity and nationalism. Ethnicities 25, 125–148. doi: 10.1177/14687968241264814

[ref58] Newman A. Donohue R. Eva N. (2017). Psychological safety: a systematic review of the literature. Hum. Resour. Manag. Rev. 27, 521–535. doi: 10.1016/j.hrmr.2017.01.001

[ref59] Ning H. (2023). Analysis of the value of folk music intangible cultural heritage on the regulation of mental health. Front. Psych. 14:1067753. doi: 10.3389/fpsyt.2023.1067753, 37065889 PMC10090295

[ref60] Owolabi H. O. Ayandele J. K. Olaoye D. D. (2020). A systematic review of structural equation model (SEM). Open J. Educ. Dev. 1, 27–39. doi: 10.52417/ojed.v1i2.163

[ref61] Pai C. K. Chen H. Lee T. J. Hyun S. S. Liu Y. Zheng Y. (2024). The impacts of under-tourism and place attachment on residents’ life satisfaction. J. Vacat. Mark. 30, 694–712. doi: 10.1177/13567667231164807

[ref62] Pan L. Xu X. A. Lu L. Gursoy D. (2021). How cultural confidence affects local residents’ wellbeing. Serv. Ind. J. 41, 581–605. doi: 10.1080/02642069.2018.1540595

[ref63] Petković J. S. (2007). Traditional values and modernization challenges in forming urban and rural culture. Facta Univ. Ser. Philos. Sociol. Psychol. Hist. 1, 23–39.

[ref64] Rouchy J. C. (2002). Cultural identity and groups of belonging. Group 26, 205–217. doi: 10.1023/a:1021009126881

[ref65] Roussin C. J. MacLean T. L. Rudolph J. W. (2016). The safety in unsafe teams: a multilevel approach to team psychological safety. J. Manage. 42, 1409–1433. doi: 10.1177/0149206314525204

[ref66] Safiih M. Azreen N. (2016). Confirmatory factor analysis approach: a case study of mathematics students’ achievement in TIMSS. Malays. J. Math. Sci. 10, 41–51.

[ref67] Sarason I. G. (2013). Social support: theory, research and applications. Berlin: Springer Science and Business Media.

[ref68] Scheepers D. Ellemers N. (2019). “Social identity theory” in Social psychology in action: evidence-based interventions from theory to practice (Cham: Springer International Publishing).

[ref69] Shandil D. N. (2024). Spiritual power of music: healing and transformation. Swar Sindhu: Natl. Peer-Rev. Refereed J. Music 12.

[ref70] Sherman D. K. Cohen G. L. (2006). The psychology of self-defense: self-affirmation theory. Adv. Exp. Soc. Psychol. 38, 183–242. doi: 10.1016/S0065-2601(06)38004-5

[ref71] Silverman M. (2022). Music therapy in mental health for illness management and recovery. Oxford: Oxford University Press.

[ref72] Singh B. Shaffer M. A. Selvarajan T. T. (2018). Antecedents of organizational and community embeddedness: the roles of support, psychological safety, and need to belong. J. Organ. Behav. 39, 339–354. doi: 10.1002/job.2223

[ref73] Swami V. Stieger S. Voracek M. Aavik T. Abdollahpour Ranjbar H. Adebayo S. O. . (2025). Life satisfaction around the world: measurement invariance of the satisfaction with life scale (SWLS) across 65 nations, 40 languages, gender identities, and age groups. PLoS One 20:e0313107. doi: 10.1371/journal.pone.0313107, 39841629 PMC11753666

[ref74] Tang K. (2021). Singing a Chinese nation: heritage preservation, the yuanshengtai movement, and new trends in Chinese folk music in the twenty-first century. Ethnomusicology 65, 1–31. doi: 10.5406/ethnomusicology.65.1.0001

[ref75] Tao C. Huang S. S. Brown G. (2020). The impact of festival participation on ethnic identity: the case of Yi torch festival. Event Manag. 24, 527–536. doi: 10.3727/152599519X15506259856156

[ref76] Tekman H. G. Hortaçsu N. (2002). Music and social identity: stylistic identification as a response to musical style. Int. J. Psychol. 37, 277–285. doi: 10.1080/00207590244000043

[ref77] Walker R. Schultz C. Sonn C. (2014). “Cultural competence–transforming policy, services, programs and practice” in Working together: aboriginal and Torres Strait islander mental health and wellbeing principles and practice (Canberra: Commonwealth of Australia).

[ref78] Wang H. (2024). Moral thought: evaluation on the moral education and virtue cultivation of traditional Chinese music. Trans/Form/Ação 47:e02400167. doi: 10.1590/0101-3173.2024.v47.n5.e02400167

[ref79] Wang Q. Bing H. Wang S. Xu Q. (2022). Study on the spatial distribution characteristics and influencing factors of famous historical and cultural towns or villages in Hubei Province, China. Sustainability 14:13735. doi: 10.3390/su142113735

[ref80] Wang Y. Thotham A. (2025). Ethnomusicological insights into the sociocultural dynamics of folk songs in southern Shaanxi, China. Orient. Anthropol. 25, 25–42. doi: 10.1177/0972558x251314333

[ref81] West S. G. Taylor A. B. Wu W. (2012). “Model fit and model selection in structural equation modeling” in Handbook of structural equation modeling. ed. Hoyle R. H. (New York, NY: Guilford Press).

[ref82] Yin J. Ji Y. Ni Y. (2023). How does cultural confidence awaken community citizenship behaviors? The moderating effect of cultural involvement. J. Hosp. Tour. Manag. 55, 425–434. doi: 10.1016/j.jhtm.2023.05.014

[ref83] Zhang L. 2017 Hubei drum and its further development from the perspective of intangible cultural heritage. In: 3rd International Conference on Arts, Design and Contemporary Education (ICADCE 2017). Dordrecht: Atlantis Press.

[ref84] Zhang X. Peng B. Zhou L. Lu C. Wang Y. Liu R. . (2024). Tourism development potential and obstacle factors of cultural heritage: evidence from traditional music in Xiangxi. J. Geogr. Sci. 34, 309–328. doi: 10.1007/s11442-024-2206-2

[ref85] Zhong L. Qi X. Sun S. Liu J. Law R. (2025). Symbolic interactionism: exploring the experience of traditional costume in a destination. Curr. Issues Tour. 28, 1100–1115. doi: 10.1080/13683500.2024.2323161

[ref86] Zhou Y. Gu H. (2025). Enhancing rural resilience through the rural revitalisation strategy in rural China: evidence from Wushi Village, Hunan Province. J. Rural. Stud. 113:103493. doi: 10.1016/j.jrurstud.2024.103493

